# Papillomaviruses and cancer: commonalities and differences in HPV carcinogenesis at different sites of the body

**DOI:** 10.1007/s10147-023-02340-y

**Published:** 2023-05-18

**Authors:** Nagayasu Egawa

**Affiliations:** Department of Pathology, University of Cambridge, Cambridge, CB2 1QP UK

**Keywords:** HPV, Cancer, Carcinogenesis

## Abstract

Human papillomavirus (HPV) is associated with 5% of all cancers globally at a range of body sites, including cervix, anus, penis, vagina, vulva, and oropharynx. These cancers claim > 400,000 lives annually. The persistent infection of HPV and the function of viral oncogenes are the primary causes of HPV-related cancers. However, only some HPV-infected persons or infected lesions will progress to cancer, and the burden of HPV-associated cancer varies widely according to gender and the part of the body infected. The dissimilarity in infection rates at different sites can explain only a small part of the differences observed. Much responsibility likely sits with contributions of specific epithelial cells and the cellular microenvironment at infected sites to the process of malignant transformation, both of which affect the regulation of viral gene expression and the viral life cycle. By understanding the biology of these epithelial sites, better diagnosis/treatment/management of HPV-associated cancer and/or pre-cancer lesions will be provided.

## Introduction

It was presumed from epidemiological facts that cervical cancer was caused by infectious factors related to sexual intercourse but for a long time the details were unknown [1]. Forty years ago, Harald zur Hausen and colleagues discovered the first link between human papillomavirus (HPV) and cervical cancer, by finding new types of HPV, HPV16 and HPV18, in cervical cancer tissues [2, 3]. Since then, studies confirmed the direct role of several mucosal HPV types, which are now categorised as high-risk HPVs, in the development of cervical cancer and other epithelial tumours, including cancers of the oropharyngeal, anus, penis, vulva, vagina, and skin [4]. The Nobel Prize in Physiology of Medicine, in 2008, was awarded to Dr zur Hausen for his discoveries that led to the expansion of the spectrum of HPV-related cancers and the understanding of the viral carcinogenesis mechanisms. The discovery that cervical cancer is a form of viral infection has had significant impact, leading to the development of vaccines to prevent HPV infection and a more accurate cervical cancer screening algorithm using HPV tests [5], which will lead us to the elimination of cervical cancer and HPV-related cancer (WHO: Cervical cancer elimination initiative. https://www.who.int/initiatives/cervical­cancer­elimination­initiative).

**Fig. 1:**
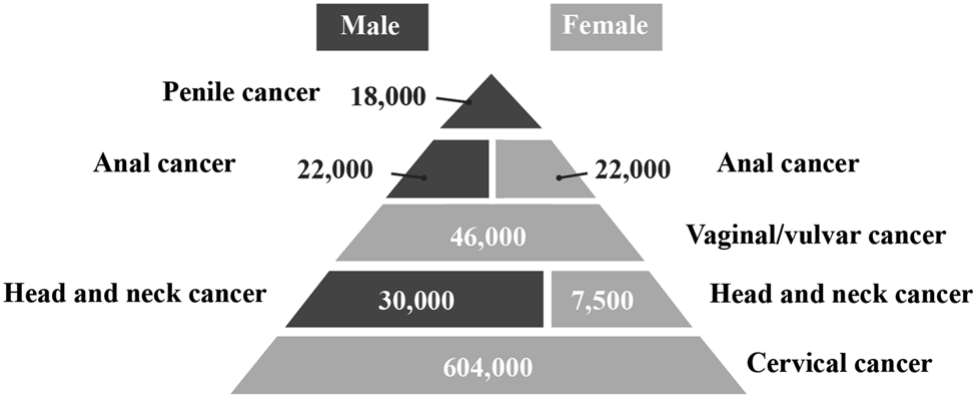
The burden of cancers attributable to HPV infection by site and gender [4, 9]

One of the most distinctive characteristics of HPV is the genotype-specific preference for distinct anatomical sites (tropism) of particular HPV types, where they cause lesions with distinctive clinical pathologies (reviewed in [6, 7]). The origin of papillomaviruses is linked to changes in the epithelium of their ancestral host that occurred at least 350 million years ago [8]. Since then, they have co-evolved as their different host species have evolved but appear not to have followed an identical evolutionary path to that of their hosts. Rather, they have paralleled the evolution of host resources or attributes, such as the presence or absence of fur or the evolution of sweat glands [7]. HPV is required to evolve distinctive viral gene functions and regulation of viral gene expression during adaptation to the sites of infection, which must have different biology. As consequence, this has resulted in the diversity of pathogenicity, HPV's genotype-specific pathogenicity. The association of “high-risk” human papillomavirus types with cervical cancer and other HPV-related cancers is now well established. The infection of high-risk HPVs causes almost 5% of human cancers worldwide, more than 700 000 cases of HPV-associated cancers, and 400 000 deaths worldwide each year (Fig. 1) [4, 9].

## HPV pathogenesis

Given the impact of high-risk HPV as a cancer causative agent, it is reasonable that the study of HPV has been driven by the association of the high-risk Alphapapillomavirus types to anogenital and oropharyngeal cancers [10]. By comparing high- and low-risk HPV, a significant role for malignant transformation is assigned to the E6 and E7 genes – often referred to as the viral oncogenes – and the function of their respective proteins. E6 and E7 are efficiently able to immortalise various human cell types in vitro when they are expressed together [11]. The only viral genes expressed in cancerous, malignant HPV-infected tissue are E6 and E7, and the proliferation of cervical cancer cells per se is dependent on the expression of E6 and E7 [12]. In terms of carcinogenicity, E6 and E7 of high-risk HPVs have been shown to have various functions that are not present in E6 and E7 of low-risk HPVs [6, 13].

**Fig. 2:**
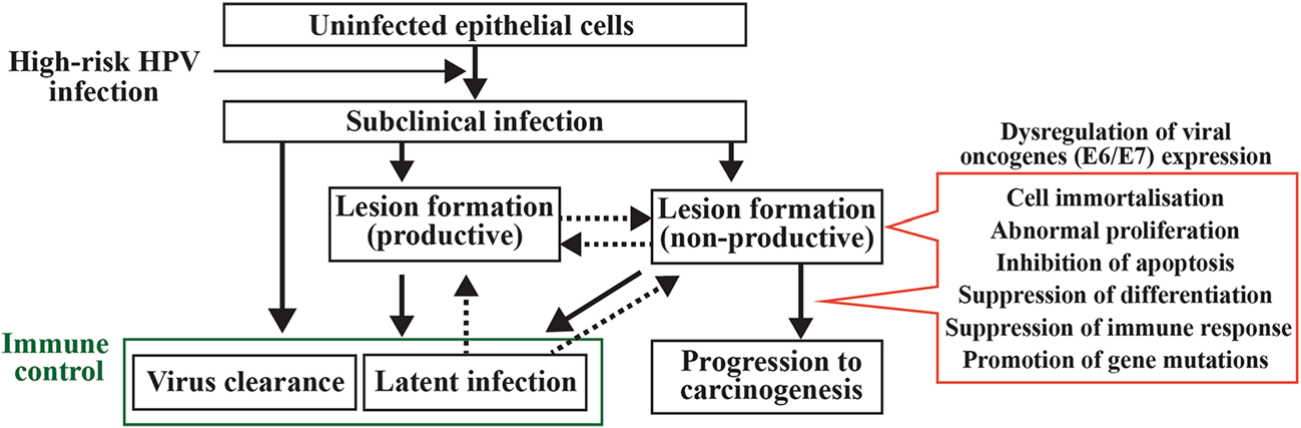
The natural history of HPV infection and its malignant transformation. Most of the infections are either asymptomatic or forming transient lesions that are eventually cleared by the function of the host's immune system. In this normal viral life cycle (productive infection), viral gene expression is optimised and well-controlled to produce virus progenies. Dysregulated expression of viral oncogenes (E6 and E7), which are represents a non-productive infection, are able to carry out many of the steps required for carcinogenesis and leads to malignant transformation in only a small proportion of persistent infections

Some of the notable functions of the high-risk E6 protein are its interaction with and degradation of TP53 and BAK, which result in the inhibition of growth arrest and apoptosis after DNA damage, as well as an accumulation of mutations [14, 15]. E6 has also been shown to interact with Myc to activate the telomerase reverse transcriptase promoter [16, 17], and degrade NFX1-91, a repressor of the hTERT promoter [18]. These changes result in increased hTERT expression and induce telomerase activity, contributing to the immortalisation of epithelial cells by maintaining telomere length. Another key difference between high-risk and low-risk papillomavirus types is the presence of the PDZ (PSD95/Dlg/ZO-1) binding motif (PBM) at the C-terminus of the high-risk E6 proteins [19]. With the PBM, high-risk E6 interacts with a range of PDZ domain-containing proteins, and, in many cases, leads to their degradation, implicating in signal transduction, cellular polarity, and transformation [20]. In addition, E6 E7 is well-known to bind to pRb, and its family members, p107 and p130, to facilitate the release of transcription factors such as E2F family members and induce the transcription of particular genes (e.g. cyclin A and cyclin E). These contribute to DNA synthesis and cell-cycle progression, counteracting the function of the cyclin-dependent kinase inhibitors p21WAF1/CIP1, p27KIP1, and p16INK4a [21]. The expression of E6 and E7 together could induce numerical and structural chromosome instability [22] via deregulation of the centrosome cycle from the loss of pRb family members through E7 [23, 24], and upregulation of Plk1 by the loss of p53 through E6 [25]. For evasion of the immune system, E6 and E7 independently counteract interferon signalling targeting cytokine-responsive IRF family members. IRF-3 activity is inhibited by E6 [26] while IRF-1 and IRF-9 (p48) activity is inhibited by E7[27, 28]. E7 also has been shown to inhibit the cGAS-STING pathway [29, 30]. Transcriptionally, E6 and E7 have been shown to inhibit the expression of STAT-1 and IFN-κ [31, 32]. As such, E6 and E7 of high-risk mucosal HPVs, if expressed, can carry out many of the steps required for oncogenesis (the hallmarks of cancer [13, 33]), including cell immortalisation, abnormal proliferation, inhibition of apoptosis, suppression of differentiation, suppression of immune response, and promotion of gene mutations (Fig. 2). In contrast, E6 and E7 of low-risk HPVs or other HPV types have been shown to have no or only inadequate forms of such functions. This difference in function well explains why high-risk HPVs cause cancer and low-risk HPVs do not.

Given the malignant transformation of the infected lesion, which produces no virus progeny, the development of cancer from lesions does not contribute to the virus fitness and is considered to be a collateral consequence of the infection [7]. Furthermore, given that low-risk HPVs can establish infectious lesions in humans without these oncogenic features, and in fact thrive more than high-risk HPVs, it is clear that the oncogenic features of high-risk HPVs were not evolved for oncogenesis, and their significance within the viral life cycle and true infectious niche, for which high-risk HPV has evolved to adapt, must be reassessed.

## Factors that affect HPV malignancy

Although high-risk HPV infections are common, with the majority of people being infected at least once in their lifetime, most will develop only subclinical or transient lesions that spontaneously regress, and invasive cervical cancer will only develop in some persistent infections/lesions. Also, it is now being accepted as the natural history of HPV infection that HPV infection is not always cleared completely but becomes a controlled infection below the limits of detection (latent infection) [34, 35], which is not considered an immediate precancer/cancer risk but contributes to, in a relatively small amount, the risk of cancer development in later life [36]. Prospective epidemiologic studies, combined with strong biological plausibility explanations derived from basic sciences, assured the concept of high-risk HPV infection as a necessary, but insufficient causative agent of almost all cervical cancers [37].

Clinically, HPV infection in the cervix is classified depending on its risk of developing precancer and cancer. For example, a subclinical infection can be HPV-positive, but no cervical abnormalities are detected. Though the detection of high-risk HPV is considered as the risk of developing precancer and cancer, the lack of detectable cervical lesions is considered as there being no immediate risk of cancer. Such subclinical infections have become more common since the HPV test was introduced as part of primary cervical cancer screening. CIN1, which is referred to as the LSIL (low-grade squamous intraepithelial lesions) designation used in the Bethesda system, also does not show a significantly higher risk of precancer lesions [38]. As there is currently no effective and non-invasive treatment for these HPV infections, patients with this condition are just placed under frequent follow-up. The majority of newly acquired HPV infections and lesions are transient and resolve in 1–2 years without intervention as a result of an effective immune response, and only a minority of HPV infections are detected persistently beyond this time span, which increases the risk of developing cervical pre-cancer lesions. CIN2 and 3, which are referred to as HSIL (high-grade squamous intraepithelial lesions), can progress to invasive cervical cancer if untreated [39]. CIN2 is an equivocal precancer lesion and is known to have some regression potential. However, in many countries this is the threshold for excisional treatment to provide a safety margin against invasive cancer risk [40]. CIN1 and CIN2 can be caused by a broader spectrum of HPV types including high- and low-risk HPV types. In contrast, CIN3 is virtually caused by only high-risk HPVs [41].

Histopathologically, the classification of CIN1-3 reflects the extent to which basal-like cells (undifferentiated cells with a high nuclear/cytoplasmic ratio and nuclear atypia) have replaced the cervical epithelium. Since the discovery that cervical dysplasia is an HPV-infected lesion, our understanding of cervical dysplasia has become much more advanced. Low-grade lesions (CIN1) typically represent a "productive infection", where the level and pattern of viral gene expression are properly controlled to complete the stages of its viral life cycle and to produce virus progeny. In contrast, high-grade lesions represent "non-productive" or "abortive infection", where the expression of viral genes is dysregulated (just two viral oncoproteins, E6 and E7, are mainly expressed) and no infectious virus is produced. Virologically, the extent to which basal-like cell replacement has occurred reflects the extent of viral oncogene expression.

The deregulated expression of high-risk E6 and E7 proteins is the primary risk of the development of cancer, and it explains the difference in the risk of developing cancer among cervical dysplasia. Deregulation of viral gene expression may be caused by viral genome integration, which is often observed in high-grade premalignant lesions and cancer [42, 43]. Such integrations disturb the viral E2 and E8^E2 ORF, which encodes a transcriptional repressor of E6/E7 mRNA expression. In addition, E6/E7 mRNA transcribed from integrated genomes has been known to be more stable [44]. Thus, E6 and E7 protein expression can be facilitated from integrated HPV genome fragments rather than episomal virus genomes. This viral genome integration can be triggered by the genomic instability caused by viral E6 and E7. Interestingly, some cervical cancers retain viral genomes in episomal form and, in these cases, high-risk E6 and E7 expression is considered to be deregulated by aberrant epigenetic modifications of the viral genome [42, 45]. It has been reported that several factors such as the type of cell, hormones, epithelial environment, host immunity, and inflammatory cytokines affect viral gene expression [46–49]] and act differently to drive malignant transformation. This could explain why different sites of the body infected with high-risk HPV have a different chance of cancer development. The incidence of cancer and HSIL is particularly high among those with HIV and other forms of immune suppression, suggesting that host immunity plays an important role in controlling HPV infection [50–52].

## Non-infectious co-factors for HPV carcinogenesis

Compared to the infection of carcinogenic high-risk HPV types, non-infectious co-factors play a minor part in cancer development. Many potential co-factors, which may affect the risk of infection or risk of persistent/dysregulated infection, have not been evaluated rigorously enough in epidemiological studies (see [53]).

(i) Tobacco smoking has shown a significant increase in the development of cervical cancer and its precursor lesions [54]. The molecular mechanisms by which smoking increases the risk are not well understood; smoking may inhibit the immune response to HPV and facilitate persistent/dysregulated infection or smoking carcinogens may accumulate DNA damage while HPV E6 blocks the apoptosis and cell cycle arrest triggered by DNA damage. Tobacco-specific carcinogens can be detected in cervical mucus [55].

(ii) The long-term use of oral contraceptives has been hypothesized to be associated with development of precancer and invasive cervical cancer. The regulatory region of HPV genome contains hormone response elements that can be stimulated by estrogen and affect the proliferative capacity of the infected cells. There is also some evidence of cooperation between estrogen and HPV in the development of cervical cancer in humans and in model systems. However, the interpretation of studies of oral contraceptives for risk of cervical cancer has been impeded by limitations in design (i.e., not taking HPV status appropriately) confounded by Pap smear history (a low prevalence of Pap smear screening resulted in increased cervical cancer risk), and the formulation and dose of hormonal contraceptives. In summary, the long-term (5 yeas o more) use of oral contraceptives can increase the risk up to twice as much [56], with limited data suggesting that the relative risk of cervical cancer associated with oral contraceptive use might decline after cessation of use.

(iii) The microbial microenvironment in cervical tissue is considered to determine the integrity of homeostasis and protective mucous layer of cervical tissue by maintaining healthy immunity and metabolic signalling. This can affect HPV infection or risk of persistent/dysregulated infection by changing viral gene expression. The role of chronic inflammation is less certain, with very limited data about Chlamydia trachomatis infection [57]. The influence of the vaginal microbiota in malignant transformation has only recently been investigated [58].

(iv) There have been numerous studies which examined the association between risk for cervical cancer and dietary intake or nutrient concentrations, including vitamin C, folate, β-Carotene, retinoic acid. Most of the studies were small and high rates of spontaneous regression of lesions hampers sufficient power to test the efficacy of the selected agents. In addition, most of these studies have methodological limitations in analysis, lack of adequate consideration of HPV infection, and confounding factors (e.g., tobacco smoking or oral contraceptive use, which can affect both malignant transformations and nutritional status). Due to these limitations in observational studies and supplementation trials, there has been no strong evidence of a role for any one micronutrient in the development of cervical cancer. Limited data, though, suggests that higher folate is possibly protective against the risk of malignant transformation.

(v) Familial clustering of cervical cancer has been explored as a potential risk marker of inherited genetic susceptibility for cervical cancer. In general, these studies failed to differentiate sufficiently the effects of genetic susceptibility from those of environmental and behavioural factors, with indications of a familial risk, and requires further investigation. As the control of HPV infection (spontaneous regression of LSILs and even HSILs) is mediated by cellular immune responses against virus-specific antigens, it is reasonable that the polymorphisms of HLA Class I and II genes lead to variations in the antigen recognition site that may confer susceptibility or resistance to HPV infection and malignant transformation. HLA recognition is considered to be HPV type-specific, and HLA polymorphisms can also explain that HPV type-specific prevalence varies according to geographic area and ethnicity. Indeed, epidemiological studies in different populations have found various relationships between risk for malignant transformation and HLA polymorphisms. However, it has not been utilised for diagnosis/treatment/management of cervical pre-cancer lesions.

## HPV carcinogenesis at the different sites of body

The primary risk of HPV-associated cancer development is a function of HPV infection. High-risk human papillomaviruses infect a wide range of epithelial sites, however, the frequency of HPV-associated cancer among the sites of bodies shows huge diversity (Fig. 1) due to the difference in the prevalence of high-risk HPV infection in each site, as well as in the different which affect persistent infection and malignant transformation. The site where HPV-associated cancers occur most frequently appears not to be the typical stratified epithelia sites that support productive infection and is also considered vulnerable to HPV infection due to the lack of a particular stratified defensive epithelial barrier to infection. In general, HPV-associated cancers are preventable by prophylactic vaccine if people get vaccinated before the first infection. The vaccines are HPV-type specific and the efficacy of the vaccine in cancer incidence would be different based on the attribution of each HPV type in cancers among the parts of the body, geographic area, and ethnicity. Cervical cancer can be caused by a relatively broader spectrum of high-risk HPV types. In contrast, other HPV-associated cancers are caused predominantly by HPV16 and HPV18, which are targeted by all available HPV vaccines.

### Cervical cancer

**Fig. 3:**
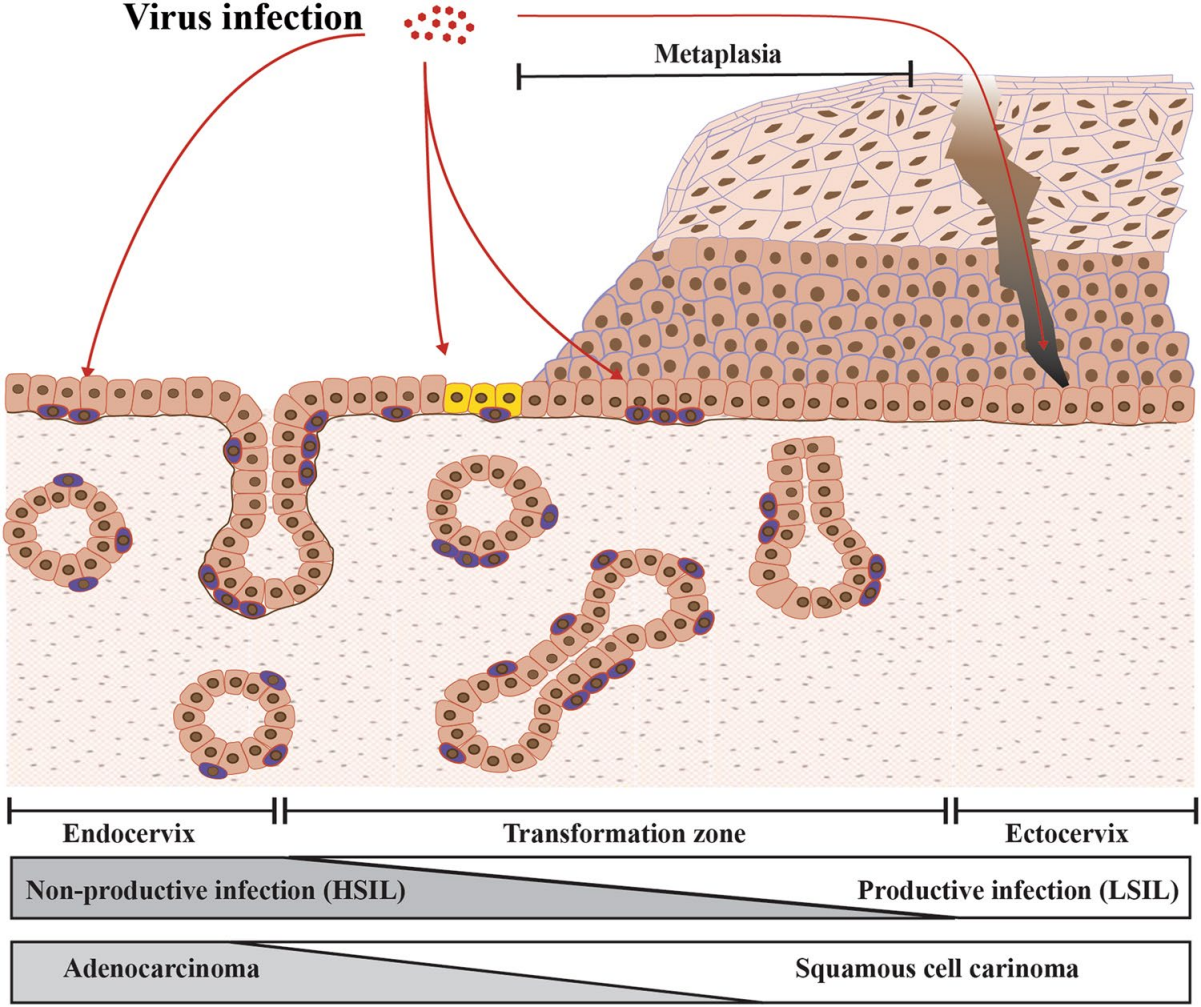
Diversity of host cells and environments affect viral pathogenicity at cervix. Most cervical cancers arise at the cervical transformation zone, where is maintained by a specialized type of tissue stem cell known as the reserve cell (shown in purple), and possibly also by a cluster of cuboidal cells (yellow) localized more precisely at the squamo-columnar junction. These cells can maintain either the columnar epithelium or the stratified epithelium (metaplasia) of transformation zone depending on their extracellular environment. The cells in the ectocervix maintain the conventional stratified epithelium, in contrast the cells in endocervix lack the ability to stratify. Current thinking suggests that the cells at the transformation zone fail to properly regulate viral gene expression, leading to a non-productive rather than a productive infection, which can be seen in ectocervix, and are more likely to progress to malignant transformation. The cells at the endocervix also fail to properly regulate viral gene expression, suggesting that high-risk HPV infection at this site might not develop an LSIL precursor, but adenocarcinoma in situ

The infection of high-risk HPV types is associated with virtually all (> 99%) cervical cancers. In some developed countries, the number of cases other HPV-associated cancers is increasing. The incidence of HPV-associated head and neck cancer is also increasing and is greater than the incidence of cervical cancer. However, cervical cancer is the most prevalent (> 80%) cancer among all HPV-associated cancers worldwide. While cervical cancer reaches over 600,000 cases per ear worldwide, HPV-associated cancer of the vulva, vagina, and penile cases reaches around 50,000 per year [4]. The cervix consists of at least three distinct epithelial types: the ectocervix (the stratified epithelium), the transformation zone, and the endocervix (the columnar epithelium) (Fig. 3). Most cervical cancer develops at the transformation zone which is the area where the glandular epithelium is being replaced by squamous epithelium (metaplasia). Metaplasia is a normal process and is induced by, upon puberty, hormonal changes that cause the cervix to evert, resulting in the exposure of columnar epithelium to the more acidic environment in the vagina. This process can also be induced by local irritation. The transformation zone is maintained by a specialised type of cell known as the reserve cell, or possibly also by a cluster of cuboidal cells, which represent the tissue stem cells that can differentiate into both stratified squamous and columnar epithelia depending on their extracellular environment. Our current model suggests that the cells at the transformation zone fail to properly regulate viral gene expression, leading to a non-productive or abortive infection rather than a productive infection (seen in ectocervix infection), and are more likely to progress to malignant transformation. The endocervix lacks the ability to stratify and is thought not to support the productive papillomavirus life cycle and viral gene expression here is deregulated, suggesting that high-risk HPV infection at this site might not develop an LSIL precursor. As such, cervical cancer appears not to be one disease, but to be heterogenous cancer with three progression routes that depend on the nature of the initially infected cell [59]). At least, our conventional model, that HPV infection leads primarily to productive infection and progresses to malignant transformation, is not always true.

### Head and neck squamous cell carcinoma (reviewed in [60, 61])

Head and neck squamous cell carcinoma (HNSCC) develops at different anatomic sites of the upper aerodigestive tract including the nasopharynx, paranasal sinuses, oral cavity, oropharynx, hypopharynx, and larynx. Since HPV was discovered in cervical cancer, HPV has also been recognised as a causative agent for HNSCC, especially for oropharyngeal squamous cell carcinoma (OPSCC). The main difference when compared with cervical cancer is that OPSCC can be observed as two (HPV-negative and HPV-positive) diseases. The socioeconomic status of patients, molecular profiles, clinical characteristics, and especially the prognosis of the tumours, tend to differ between HPV-positive and HPV-negative OPSCC. High-risk HPV-associated OPSCC makes up a substantial proportion of all OPSCC cases and this number is rising, mainly in the Western world. This is most likely due to changes in sexual behaviour [62, 63]. The significant gender differences in the prevalence of HPV positive tumours could be explained by the differences in rates of HPV transmission between vaginal–oral and penile–oral sex, however HPV is the major driver of OPSC in both sexes in the USA (62% of male and 56% of female OPSCC)[64]. In contrast, lower socioeconomic status and poor oral hygiene are related to HPV-negative OPSCC. HPV-negative OPSCC also possesses genomic complexity and very frequent alterations in the tumour suppressor TP53 and in cell-cycle regulators, which are often intact in HPV-positive OPSCC. These molecular differences might result in low susceptibility to treatments (radiation and anticancer drugs) and unfavourable prognosis of HPV-negative OPSCC patients.

HPV infection is also associated with a small number of other HNSCCs, however, the contribution of each site varies hugely (e.g., HPV infections are more common in the oral cavity but HPV-associated oral cavity squamous cell carcinoma (OCSCC) accounts for only 4% of cases, while in contrast, 47% of cases of OPSCC is HPV-associated). HPV-positive OPSCC often arises from the tonsillar crypt epithelium. Interestingly, reticular tonsillar crypt epithelium would provide easier access to HPV than other stratified squamous epithelium, and it suggests that its malignant transformation reflects the different biology of infected tissue at each anatomical site. The tonsillar reticular crypt epithelium might not be permissive for productive HPV infections that support the viral life cycle to generate viral progeny, but may be permissive for abortive/non-productive infection, where viral gene expression is dysregulated and likely to progress to malignant transformation (like the cells described at the cervical transformation zone). This is consistent with the observations that large studies on non-malignant tonsil samples have detected no or low HPV prevalence, and LSIL-type productive lesions, which are the most common infected lesion at cervix, have not been identified as a cancer precursor at the tonsillar crypt.

### Anal cancer

High-risk HPV has been detected in 90% of anal cancers with HPV16 detected in 80% of those cases [4, 65]. The incidence of anal cancer has been rising among both women and men in the general population since the 1970s. This is primarily attributable to changes in sexual behaviour resulting in an increasing trend of HPV infection following the "Sexual Revolution" in the late 1960s and 1970s [62]. Similar to the cervical transformation zone, the anal transformation zone, where stratified epithelium abuts columnar epithelium, is a target site of HPV infection and may have a type of cell which is favourable to abortive infection. Anal HPV prevalence varies substantially by HIV-status and sexual orientation (especially in men), which is considered to increase HPV prevalence at the anal site. In women, there is also a clear relationship between history of cervical or vulvar HSIL/cancer and anal cancer, which may also result in increased probability of high-risk HPV infection at the anal site. Altogether, anal cancer is considered to share similarities in its biology and natural history with cervical cancer, including association with high-risk HPV infection to precancerous lesions to cancer. Recently, it was shown that, amongst patients with historically proven anal HSIL, the risk of development of anal cancer was significantly decreased by treatment for anal HSIL compared to patients with only active monitoring [66]. So far, the results support the benefits of screening and treatment for anal HSIL, like cervical HSIL, as the standard of care for persons living with HIV who are over 35 years old. However, it may be relevant for other groups at increased risk for anal cancer as well.

### Vulvar and penile cancer

Two distinct pathways to invasive squamous cell carcinoma (SCC) exist in the vulva with HPV being detected in one-third of vulvar SCC [67]. This difference appears even in its precursor lesions, HSIL and dVIN (differentiated vulvar intraepithelial neoplasia) respectively. HPV-positive vulval cancers and precursors are with younger age and smoking. In contrast, HPV-negative vulval cancer and precursor lesions are related to older age and chronic inflammatory disorders such as Lichen Sclerosus (LS). Histopathologically, HSIL represents a proliferation of immature basaloid cells with a high nuclear/cytoplasmic ratio that replaces most of the epidermis. dVIN represents an epidermis that is thickened, where keratinocytes have increased glassy cytoplasm. Clinically, while dVIN comprises a minority (up to 10%) of all precursor lesions, it is found in a majority (36–79%) of SCC. The likelihood of progressing to cancer is higher for dVIN than for HSIL. The development of penile cancer is also shown to have two pathways to malignant transformation, with the presence or absence of HPV infection. Similar to vulvar cancer, a majority (up to 90%) of penile cancer precursor, penile intraepithelial neoplasia (PeIN), is HPV-positive with HPV 16 being the most common type (40%). In contrast, a lower number (50%) of penile carcinomas is associated with HPV. HPV-negative penile cancer and precursor lesion are, again, related to older age and phimosis and/or chronic inflammation, such as Lichen Sclerosus. The prevalence of HPV infection in adult men and women appears to be high across age groups but the incidence of HPV-associated vulvar and penile cancer is far lower than the incidence of cervical cancer, suggesting that the vulvar and penile epithelium might be more permissive for productive HPV infections that support the viral life cycle to generate viral progeny with proper control of viral gene expression (or be more likely to be controlled by host-immunity) but not abortive/non-productive infection which is more likely to progress to malignant transformation. These principles would also apply to vaginal cancer.

## Conclusion

High-risk human papillomavirus infection causes HPV-associated cancer, with a great diversity of its incidence among the sites of the body infected. It appears that differences in infection rates at different sites are only a small part of the reason for this. Specific epithelial stem cells and the cellular microenvironment at these sites must be influencing the control of viral gene expression and contributing differently to the process of malignant transformation. In contrast to our understanding of high-risk HPV protein functions, our knowledge of the biology/molecular mechanisms of the different epithelial sites is very limited, and further studies are required to provide better diagnosis/treatment/management of HPV-associated cancer and/or pre-cancer lesions.
